# Thermal properties of nanofluids using hydrophilic and hydrophobic LiYF_4_:Yb/Er upconverting nanoparticles†

**DOI:** 10.1039/d3na01114c

**Published:** 2024-02-14

**Authors:** João M. Gonçalves, Ana R. N. Bastos, Sidney J. L. Ribeiro, L. D. Carlos, Ricardo L. Longo, José Maurício A. Caiut, Rute A. S. Ferreira

**Affiliations:** a Department of Physics, CICECO – Aveiro Institute of Materials, University of Aveiro Aveiro 3810-193 Portugal rferreira@ua.pt; b Department of Chemistry, Faculdade de Filosofia, Ciências e Letras, University of São Paulo Ribeirão Preto 14040-900 Brazil caiut@ffclrp.usp.br; c Institute of Chemistry, Universidade Estadual Paulista «Júlio de Mesquisa Filho» Araraquara 14800-060 Brazil; d Departamento de Química Fundamental, Universidade Federal de Pernambuco Recife PE 50740-540 Brazil ricardo.longo@ufpe.br

## Abstract

Luminescent nanoparticles have shown great potential for thermal sensing in bio-applications. Nonetheless, these materials lack water dispersibility that can be overcome by modifying their surface properties with water dispersible molecules such as cysteine. Herein, we employ LiYF_4_:Er^3+^/Yb^3+^ upconverting nanoparticles (UCNPs) capped with oleate or modified with cysteine dispersed in cyclohexane or in water, respectively, as thermal probes. Upconversion emission was used to sense temperature with a relative thermal sensitivity of ∼1.24% K^−1^ (at 300 K) and a temperature uncertainty of 0.8 K for the oleate capped and of 0.5 K for cysteine modified NPs. To study the effect of the cysteine modification in the heat transfer processes, the thermal conductivity of the nanofluids was determined, yielding 0.123(6) W m^−1^ K^−1^ for the oleate capped UCNPs dispersed in cyclohexane and 0.50(7) W m^−1^ K^−1^ for the cysteine modified UCNPs dispersed in water. Moreover, through the heating curves, the nanofluids' thermal resistances were estimated, showing that the cysteine modification partially prevents heat transfer.

## Abstract 2

Luminescent nanoparticles have shown great potential for thermal sensing in bio-applications. The nanofluids thermal resistances were estimated, showing that the cysteine modification partially prevents heat transfer.

## Graphical abstract

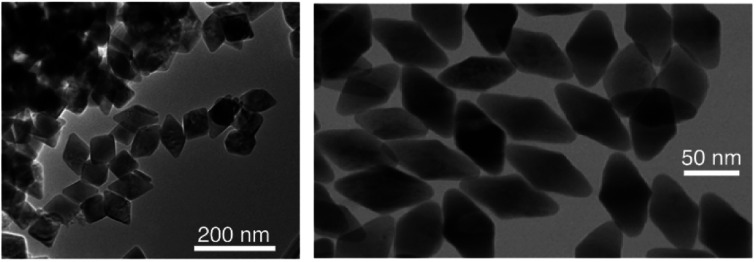

## 1. Introduction

Temperature is a key quantity for describing either natural or engineered systems, regardless of their scale.^1^ Recent technologies involving, *e.g.*, microelectronics,^2–4^ the Internet of Thing,^5^ e-Health,^6^ and intracellular processes^7,8^ strongly rely on nanoscale, which imposes a challenge in measuring temperature, because conventional thermometry cannot be used at the submicron scale, like intracellular temperature fluctuations and microfluidics.^3,8,9^ For this reason, thermometers working at the nanoscale have been investigated in recent years,^9^ with special attention to luminescent nanothermometers.^3,8–12^

Luminescence nanothermometers exploit the temperature-dependent luminescence of a thermally imaged object, yielding high relative thermal sensitivity (>1% K^−1^) and spatial resolution (<10 μm).^3^ Several types of luminescent nanothermometers have been recently developed for this specific purpose such as green fluorescent protein,^13,14^ small organic molecules,^15^ quantum dots,^16^ polymers,^17,18^ polymer dots^19^ and lanthanide-doped nanoparticles.^8,20,21^ Amongst these promising materials, lanthanide ion (Ln^3+^) based nanomaterials show several unique features and advantages, such as absence of photodegradation, high thermal and chemical stability, and due to the shielding of 4f-electrons and forbidden nature of their 4f–4f transitions,^10,22,23^ Ln^3+^ show very narrow emission bands (∼100 cm^−1^) and long luminescent lifetimes (in the order of ms).^24^ Such materials are also widely employed in energy conversion processes, particularly, upconversion (UC) emission, which converts photons in the near infrared region into visible radiation.^25^ These and other remarkable features make Ln^3+^ based materials specially suitable for biological applications, because one can avoid background fluorescence using time-resolved emission spectroscopy, and use the UC to modulate absorption into biological windows.^26,27^ For instance, Yb^3+^-doped nanoparticles are excited at 980 nm, which is located in the first biological window, attracting the attention for several biological applications.^28–31^ Usually, Er^3+^ is employed as a dopant for thermal sensing, in these Yb^3+^-doped nanoparticles, because it presents two thermally coupled levels, ^2^H_11/2_ and ^4^S_3/2_, which follows the Boltzmann distribution. This temperature dependence is due to a suitable energy separation (∼700 cm^−1^) between the barycenter of these two levels. The rate of equilibration of these two states is on the order of 10^12^ s^−1^, which then dominates over the radiative, non-radiative, and energy transfer rates.^1,3,32^ Thus, these nanothermometers can be characterized by a well-establish state equation (Boltzmann statistics), which relates the intensity ratio between the ^2^H_11/2_ → ^4^I_15/2_ and ^4^S_3/2_ → ^4^I_15/2_ transitions and the absolute temperature, providing a primary thermometer.^33^

Several materials have been explored for their UC properties, such as oxides^34,35^ and glasses,^36,37^ but special attention has been given to lanthanide-doped fluoride upconverting nanoparticles (UCNPs), due to their low phonon energy and high UC efficiency.^23^ In fact, different fluoride UCNPs have been explored in the literature such as NaYF_4_,^38–40^ LiYF_4_ (ref. 41–44) and NaGdF_4_.^45,46^ However, one of the main disadvantages of these materials is their lack of water dispersibility in some synthetic routes. To overcome this limitation, the UCNPs can be encapsulated in silica^28,47^ or by performing ligand exchange.^48,49^ Alternatively, it is also possible to modify their surface by coating with an organic layer (*e.g.*, oleic acid), which can then be modified using a variety of chemical reactions. For instance, the functional groups (*e.g.*, carboxylate, alkene) of the oleate layer in capped LiYF_4_ nanocrystals can be used for reactions with modifying agents to achieve water dispersibility, biocompatibility, targeting, *etc.* Indeed, cysteine is an amino acid with three different functional groups (–NH_2_, –COOH, and –SH) that provides a wide range of reactions, from the formation of disulphide bonds^50^ to click chemistry.^51^ Cysteine is also known to render water dispersibility to otherwise hydrophobic nanoparticles.^52^ Hence, we propose to modify oleate capped LiYF_4_ nanocrystals by reacting with cysteine, because all reagents are promptly available and highly versatile.

An important issue is whether the addition of an organic coating, to functionalize the nanoparticle for biocompatibility, water dispersibility, and targeting, impacts its ability to accurately sense the local temperature, and if heat transfer from the external environment reaches the nanoparticle to establish thermal equilibrium. To date, the thermal conductivity and the thermal resistance are often measured using experimental electric methods (*e.g.* 3ω-method and null-point scanning thermal microscopy), which are complex, expensive and had limitations for nanomaterials.^53^ UC thermometry has been recently used to overcome this issue by irradiating the sample with a laser beam leading to a temperature increase. With the dependence of the maximum temperature increase with the laser power density, it is possible to estimate the thermal conductivity, as shown in phospholipid capped LiYF_4_ nanocrystals where the thermal conductivity of a lipid bilayer was calculated.^32^ By analysing the temporal evolution of the heat dissipation (transient regime), the transient time can be determined to provide estimates of other thermal properties such as thermal resistance.^54^ This approach has been used to estimate the thermal resistances of a powder KLu_0.94_Ho_0.01_Tm_0.05_(WO_4_)_2_ (ref. 53) in contact with air, and free-standing films of Er^3+^/Yb^3+^ co-doped GeO_2_–Ta_2_O_5_ particles dispersed in poly(methyl methacrylate).^54^ Both steady-state and transient regimes provided an easy access to thermal parameters (*e.g.*, thermal conductivity, heat transfer coefficient, thermal resistance, and thermal capacity) with the advantage of being independent of the sample electrical conductivity.

Herein, we synthesised oleate capped LiYF_4_:Er^3+^/Yb^3+^ UCNPs and performed epoxidation of oleic acid followed by reaction with cysteine to provide water dispersibility for the cysteine modified UCNPs. Thus, two different nanofluids were investigated as luminescent thermal probes: a cyclohexane dispersed-oleate capped LiYF_4_:Er^3+^/Yb^3+^ UCNPs and water dispersed-cysteine modified LiYF_4_:Er^3+^/Yb^3+^ UCNPs. Both nanofluids function as primary nanothermometers within the temperature range of 290 to 320 K, displaying a notable relative thermal sensitivity and temperature uncertainty. To precisely determine the effect of the cysteine modification on thermal properties of the UCNPs, the thermal conductivities of the nanofluids were obtained from the steady-state regime of the heating curves, and their thermal resistances were estimated using the transient regime. The optical heating and temperature sensing properties of these nanofluids can potentially provide information regarding the thermophoresis (Soret effect) of the nanoparticles.

## 2. Experimental section

### 2.1. Materials

Y_2_O_3_, Yb_2_O_3_, Er_2_O_3_, trifluoroacetic acid, lithium trifluoroacetate (LiTFA), oleic acid, 1-octadecene, cysteine, HAuCl_4_, sodium citrate and solvents were all purchased from Sigma Aldrich and were used without further purification.

### 2.2. Synthesis of LiYF_4_:Er^3+^/Yb^3+^ UCNPs

LiYF_4_ nanoparticles doped with Er^3+^ (0.025%) and Yb^3+^ (3%), LiYF_4_:Er^3+^/Yb^3+^ UCNPs, were synthesized *via* thermal decomposition as described in the literature.^42^ The procedure was performed in two steps. First, the lanthanide trifluoroacetate salts, Ln(TFA)_3_, were obtained. Diluted trifluoroacetic acid (50% v/v in MilliQ water) was added to the Ln_2_O_3_ in a round-bottom flask and heated to 80 °C under reflux until a transparent solution was obtained, then it was dried at the same temperature. In the second step, 4 mmol of LiTFA, 3.879 mmol of Y(TFA)_3_, 0.120 mmol of Yb(TFA)_3_, 0.001 mmol of Er(TFA)_3_, 40 mL of oleic acid and 40 mL of 1-octadecene were mixed in a three-neck round-bottom flask and heated to 110 °C in Ar atmosphere for 30 minutes in order to remove residual water and oxygen. After this time, the mixture was heated to 300 °C for 1 hour under Ar atmosphere. After reaching room temperature, the particles were precipitated using a mixture of hexane : acetone (1 : 4 v/v) and the precipitate was centrifugated at 3600 rpm for 10 minutes. The resultant powder was washed 3 times using the same hexane:acetone mixture. The LiYF_4_:Er^3+^/Yb^3+^ UCNPs were dispersed in cyclohexane with a concentration of 4.0 mg mL^−1^.

### 2.3. Cysteine modification

The cysteine modification was performed by epoxidation of oleic acid followed by reaction with cysteine.^52^ First, approximately 18 mg of UCNP and 0.01 mmol of 3-chloroperbenzoic acid were dispersed in 4 mL of cyclohexane and 2 mL of dichloromethane and heated to 40 °C under reflux for 3 h. Then it was cooled down to room temperature, 0.2 mmol of l-cysteine were added, heated again to 40 °C and let it react for 5 h. The resulting nanoparticles were isolated *via* centrifugation at 3600 rpm for 10 minutes, washed 2 times with ethanol, and twice more with distilled water to remove impurities. The cysteine modified LiYF_4_:Er^3+^/Yb^3+^ UCNPs were dispersed in distilled water with a concentration of 4.0 mg mL^−1^.

### 2.4. Transmission electron microscopy

The images of LiYF_4_:Yb^3+^/Er^3+^ UCNPs were collected using a JEOL JEM-2100 microscope operating at 200 keV. The oleate capped UCNPs were prepared by dispersing the powder sample in dichloromethane, whereas the cysteine modified UCNPs were dispersed in ethanol. Then, one drop of the dispersions was placed onto a grid followed by evaporation of the solvent. The average sizes were calculated over 300 particles.

### 2.5. Visible-NIR absorption spectroscopy

Visible and NIR absorption spectra were recorded at room temperature, using a dual-beam spectrometer Lambda 950 (PerkinElmer) with a 150 mm diameter Spectralon integrating sphere over the range 400–1100 nm with a resolution of 1.0 nm. The baseline was recorded with two 10 mm path-length quartz cuvettes (2 polished windows) containing the reference solvent (water or cyclohexane). The molar extinction coefficient was estimated from the Lambert–Beer law.

### 2.6. Photoluminescence

A Hellma Analytics quartz cuvette (101-40-10) was used to perform the photoluminescence measurements. It was placed into a cuvette holder with a temperature controller from Quantum Northwest. For the irradiation of the UCNPs, a BrixX 980-1000 HD laser peaking at 980 nm was focused through an optical lens (7.5 cm focal distance). The laser power density was quantified as reported in Bastos *et. al.*^32^ using a power meter (FieldMaxII-TOP OP-2 Vis, Coherent), a CCD camera (BC106N-VIS/M, Thorlabs) and neutral density filters (NE10B-B, NE13B-B and NE20B-B, Thorlabs).

The spectrum acquisition was performed using a MAYA Pro 2000 (Ocean Optics) portable spectrometer connected to an optical fibre with an integration time of 2 s. Temperature-dependent fluorescence emission spectra were obtained in a temperature range between 290 and 320 K. For the calibration measurements, the temperature was increased using a Peltier-based temperature-controlled cuvette holder (TLC 50, Quantum Northwest) with accuracy of ±0.2 °C, and recorded using an immersed thermocouple (0.1 °C accuracy, K-type, Pico Technology TC-08). To ensure that the solutions reached the steady-state temperature, a time interval of 300 s was allowed between consecutive temperature measurements.

### 2.7. Dynamic temperature measurements

The nanofluids were irradiated by a pulsed laser (BrixX 980-1000 HD) at 980 nm with power densities ranging from *ca.* 65 to 250 W cm^−2^. During the heating regime, the nanofluids were irradiated for 600 s with a high-pulsed frequency of 1.5 MHz equivalent to a continuous irradiation. The resulting temperature increase was measured over time with an immersed thermocouple (K-type, 0.1 K accuracy, Pico Technology TC-08) placed at a fixed position (0.5 cm) from the excitation beam. The emission spectra of the nanofluids were recorded with the above-mentioned portable spectrometer using an integration time of 4 s.

## 3. Results and discussion

**Fig. 1:**
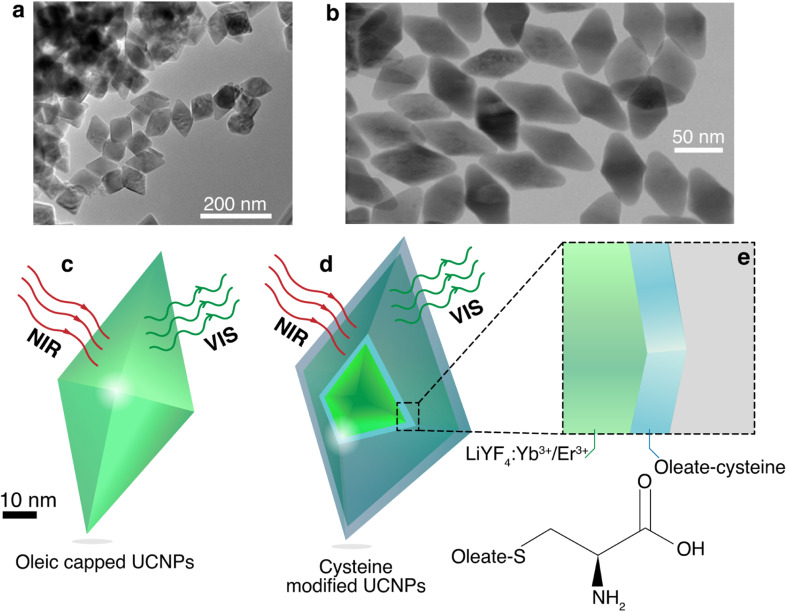
TEM images of (a) the oleate capped LiYF_4_:Er^3+^/Yb^3+^ UCNPs and (b) cysteine modified LiYF_4_:Er^3+^/Yb^3+^ UCNPs. Schematic representations of the (c) oleate capped and (d) cysteine modified UCNPs. (e) The magnification depicts a simplified 1D model for the cysteine coating, and the chemical structures of the cysteine.

Oleate capped Yb^3+^/Er^3+^ doped LiYF_4_ nanoparticles were synthesized using the thermal decomposition method,^42^ and dispersed in cyclohexane. These UCNPs presented narrow peaks in X-ray diffraction pattern (Fig. S1, ESI†) consistent with the LiYF_4_ tetragonal phase,^42^ and presented a diamond-shaped with an average size of 94 ± 15 nm (longer diagonal) and 65 ± 6 nm (shorter diagonal) with an aspect ratio of 1.4 (Fig. 1a), determined from transmission electron microscopy (TEM). To provide water dispersibility for the UCNPs, the oleate was modified *via* epoxidation followed by reaction with cysteine. The modified UCNPs maintained the same morphology with an average size of 85 ± 12 nm (longer diagonal) and 46 ± 5 nm (shorter diagonal) with an aspect ratio of 1.8 (Fig. 1b). This decrease of the average size of the nanoparticles upon modification could be due to the chemical reaction at oleate layer that might induce its tightening and compression. To ascertain the successful cysteine modification, Fourier Transform Infrared spectra (Fig. S2, ESI†) were obtained, showing new bands related with the functional groups of the cysteine amino acid, such as –NH_2_ or –NH_3_ rock at 1146 cm^−1^, N–C stretch at 1208 cm^−1^, and –CO_2_ asymmetric stretch at 1680 cm^−1^.^52,55^

The absorption spectra of the nanofluids (Fig. S3, ESI†) were measured and the corresponding molar extinction coefficients at 980 nm were estimated to be 5.7 ± 0.2 and 14.3 ± 0.1 L mol^−1^ cm^−1^ for the oleate capped and cysteine modified nanoparticles, respectively (eqn (S2), ESI†). The absorption cross section was also determined at 980 nm, yielding values of (9.3 ± 0.4) × 10^−15^ and (2.3 ± 0.1) × 10^−14^ cm^2^ for the oleate capped and cysteine modified nanoparticles, respectively, which are in same order of magnitude of similar UCNPs.^32,33^ This increase of *ca.* 2.5-fold in the absorption may be explained by the increase of the number of high energy oscillators such as N–H and O–H from the amino acid as well as the increase of the hydration shell due to the surface becoming more hydrophilic upon modification.

**Fig. 2:**
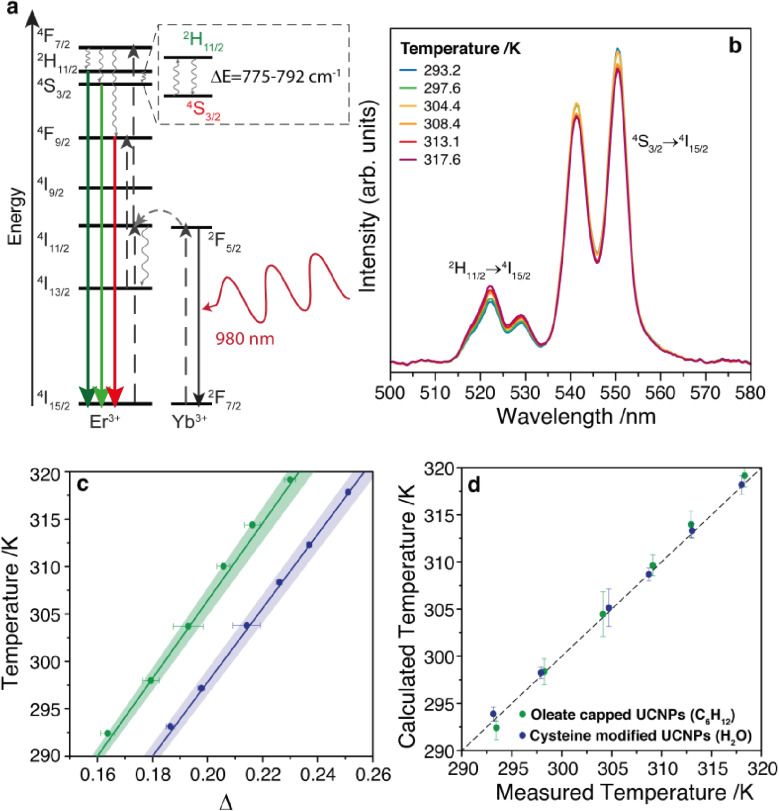
(a) Simplified energy level diagram showing energy transfer processes and main upconversion emissions in Yb^3+^/Er^3+^ co-doped nanoparticles. Upward black arrows represent absorption, the grey sideways arrow represent Yb^3+^-to-Er^3+^ energy transfer, grey wavy arrows represent nonradiative decays, and the downward green and red arrows represent the radiative Er^3+^ emissions. (b) Temperature-dependence of the upconversion emission spectra for cysteine modified UCNPs dispersed in water. (c) Temperature dependence of the thermometric parameter for oleate capped particles dispersed in cyclohexane (green) and cysteine modified particles dispersed in water (blue). (d) Comparison of the predicted temperature using the luminescence thermometer and the reference temperature measured by a thermocouple immersed into the UCNP suspension in cyclohexane (green dots) and in aqueous solution (blue dots). Dashed line represents the function *y* = *x*. All these measurements were performed with a laser at 980 nm and power density of 172 W cm^−2^.

The typical Er^3+^ UC emission spectrum was observed upon excitation at 980 nm, as depicted in Fig. 2a and b and S5, ESI.† Two transitions were observed in the green region (between 500 and 575 nm) assigned to the ^2^H_11/2_ → ^4^I_15/2_ and ^4^S_13/2_ → ^4^I_15/2_ transitions, Fig. 2a, and one ^4^F_9/2_ → ^4^I_15/2_ transition in the red region (between 675 and 700 nm),^38,44,56^ Fig. S5, ESI.† The ratio between the integrated intensity between the green and red emissions (Fig. S5, ESI†) is 1.44 for the oleate capped particles dispersed in cyclohexane and 1.07 for the cysteine modified particles dispersed in water. This slight enhancement of the red emission relative intensity in water suspensions was also observed in other nanoparticulated systems,^57^ and it was attributed to an increase of the nonradiative transitions from the upper green emitting levels ^2^H_11/2_ and ^4^S_3/2_ to the lower red emitting level ^4^F_9/2_ due to the high energy oscillators present in aqueous environment.^57,58^

The dependence of the UC spectra with temperature was investigated from 293 to 317 K (Fig. 2b and S6, ESI†). The thermometric parameter *Δ*, defined as the intensity ratio between ^2^H_11/2_ → ^4^I_15/2_ and ^4^S_13/2_ → ^4^I_15/2_ transitions, was determined and shows a clear linear dependence with the temperature, Fig. 2c. This linear behavior, with a positive slope, can be obtained from a Boltzmann-based thermometer when considering a small temperature range compared to the central temperature (24/305 = 0.079).^59^ Adopting the primary thermometer strategy reported previously,^1,3^ the temperature of the nanofluids was determined using *Δ* and eqn (S9) (ESI),† with the values for Δ*E* obtained from the emission spectra (Fig. S7, ESI†), the intensity ratio in the limit of low excitation power, and the corresponding temperature (Section IV and Table S1, ESI†). Fig. 2d shows a very good agreement between the predicted temperature using the *Δ* values and the measured temperature using a thermocouple immersed into the nanofluid, yielding a thermal sensitivity at 300 K of 1.24% K^−1^ for both nanofluids, and a thermal uncertainty of 0.8 and 0.5 K for the oleate capped particles dispersed in cyclohexane and cysteine modified particles dispersed in water, respectively (Fig. S8, ESI†), which is in agreement with values found in the literature for Yb^3+^ and Er^3+^ doped UCNPs.^1,32^

**Fig. 3:**
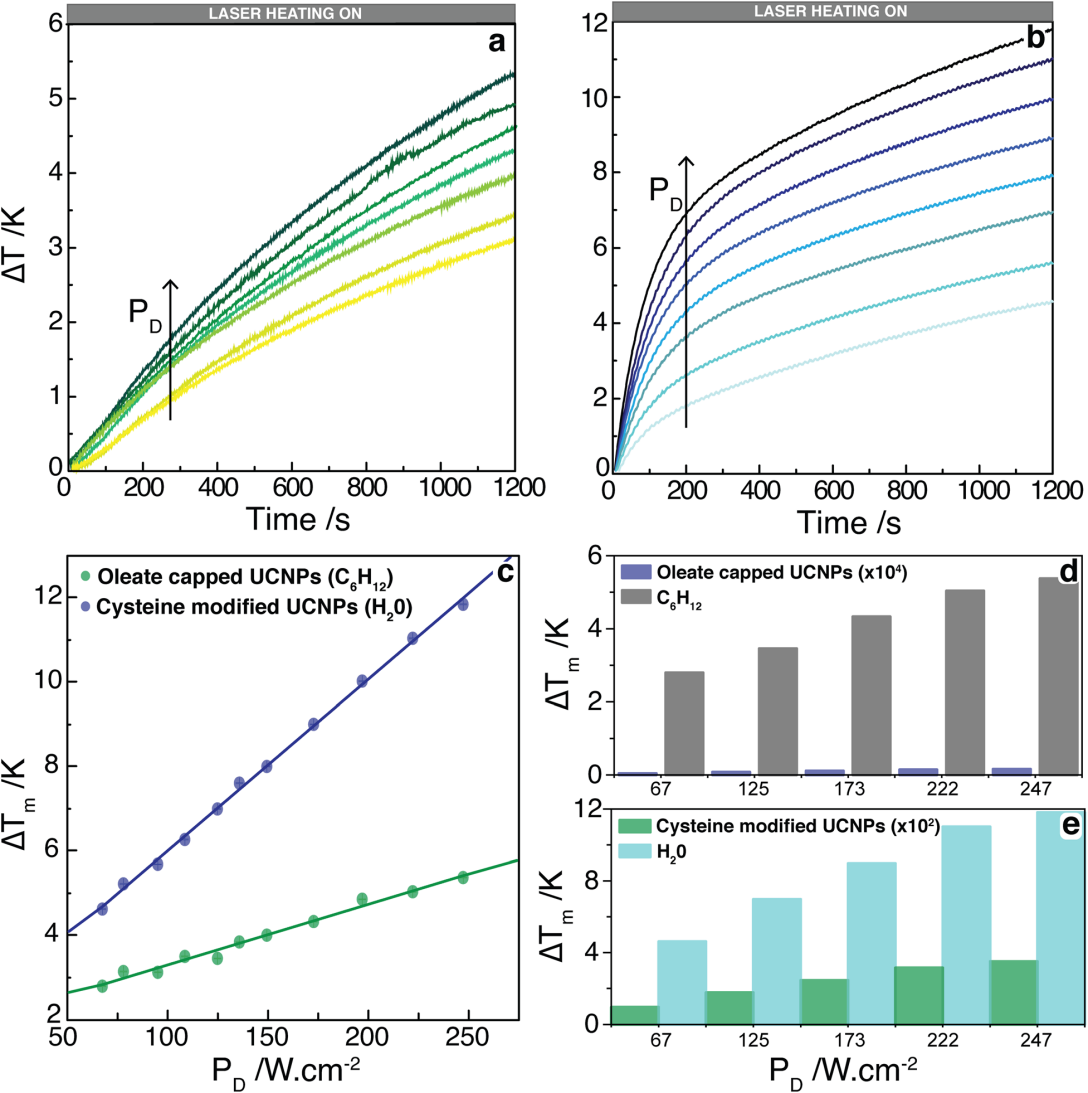
Temporal dependence of the temperature increase induced by a laser beam at 980 nm and power densities from 67 to 247 W cm^−2^ for the (a) oleate capped UCNPs dispersed in cyclohexane and (b) cysteine modified UCNPs dispersed in water. Temperature was measured by a thermocouple immersed in the solution. (c) The temperature increase at 1200 s, Δ*T*_m_, for both nanofluids as function of the laser power density. The line corresponds to the best linear fit (*R*^2^ > 0.98). (d and e) Contribution of each heat source (solvent, UCNPs) to the maximum temperature increase induced by laser excitation as function of the laser power density, measured by the immersed thermocouple. For better visualization, the contribution of the oleate capped UCNPs was multiplied by 10^4^ whereas that of the cysteine modified UCNPs was multiplied by 10^2^.

Upon irradiation at 980 nm, it can be observed a transient heating curve of the nanofluids that was recorded with a thermocouple as the temperature increase with time, Fig. 3a and b. Simultaneously, during the heating regime, the emission spectra were recorded to compare the temperature increase profiles recorded with the immersed thermocouple and the one calculated using the emission spectra. Within the experimental uncertainty of both measurements, the temperature values were similar for both nanofluids (Fig. S9, ESI†). It can also be observed that, for the same laser power density, the temperature increase is significantly higher on the cysteine modified UCNPs dispersed in water in comparison to the oleate capped UCNPs dispersed in cyclohexane. This can be explained by the ∼2.5 times increase of the absorption cross section of the UCNPs upon modification with cysteine as well as the stronger absorption of water compared to cyclohexane. To better understand this effect, the dependence of Δ*T* with the laser power density (*P*_D_), on the presence or absence of a cysteine, and on the solvent used was analyzed. From the maximum temperature increase (Δ*T*_m_) recorded at steady-state, it is possible to determine the thermal conductivities of the nanofluids.^32^ If both the solvent and the UCNPs contribute to the temperature increase in the nanofluid independently, eqn (1) can be used to model Δ*T*_m_ as function of *P*_D_, where the first term refers to the heating by the solvent, and the second term represents the heating promoted by the UCNPs,1
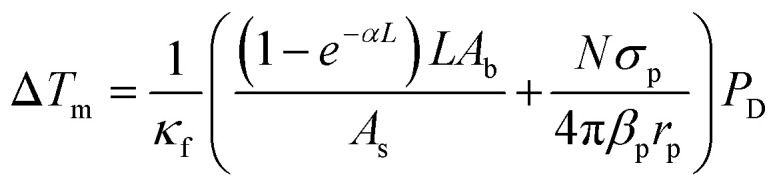
where *κ*_f_ is the thermal conductivity of the nanofluid, *α* is the solvent absorption coefficient at 980 nm, *L* is the laser path length, *A*_b_ is the area of the laser beam spot, *A*_s_ is the cross-sectional area of the heat flux, *N* is the number of UCNPs exposed to the laser beam, *σ*_p_ is the nanoparticle absorption cross-section at 980 nm, *β*_p_ is the nanoparticle geometrical correction factor due to its faceted structure,^60^ and *r*_p_ is the equivalent radius of a sphere with the same volume as the nanoparticle. All these values are presented in the Table S2, ESI.† A clear linear dependence on the Δ*T*_m_ with *P*_D_ was observed for Δ*T*_m_ determined at 1200 s, Fig. 3c. From the fit of the data with eqn (1), it was possible to estimate the thermal conductivity of the nanofluids because all quantities within parenthesis in eqn (1) can be obtained separately. The measurements performed in the oleate capped UCNPs dispersed in cyclohexane yield a thermal conductivity of 0.123 ± 0.006 W m^−1^ K^−1^, showing that the addition of these UCNPs in cyclohexane led to a small enhancement (*ca.* 5%) in the thermal conductivity compared to pure cyclohexane (0.1176 W m^−1^ K^−1^ at 300 K).^61^ This increase of the thermal conductivity in the addition of UCNPs in a solvent was expected and is in line with already reported values for nanofluids with similar and metallic nanoparticles.^32,62^ For the cysteine modified UCNPs dispersed in water, the calculated *κ*_f_ was 0.50 ± 0.07 W m^−1^ K^−1^, which is practically identical to the reported values for pure water (0.546–0.630 W m^−1^ K^−1^ at the temperature range of 290 K to 320 K).^63^ This insignificant change of the thermal conductivity of the water suspended UCNPs may be attributed to a higher contribution of the water in the conversion of NIR radiation to heat compared to the cyclohexane. Thus, for UCNPs dispersed in water, the relative contribution of the nanoparticles to the thermal properties of the system is very small, and, consequently, the thermal conductivity of the nanofluid does not increase. The calculated error in the thermal conductivity is dominated by the first term in eqn (1), which has a small uncertainty compared to the second term that depends on the number of UCNP in the laser beam and their absorption cross-section, which have large uncertainties. Hence, the estimated error in the thermal conductivity is small as reported.

The separate contribution of the solvent and the nanoparticles on the total heating of the system was calculated using eqn (1) and is presented in Fig. 3d. It can be observed that the solvents, water, or cyclohexane, were responsible to almost the totality of the thermal properties of the system. Moreover, the cysteine modified UCNPs was responsible for a higher increase of temperature than the oleate capped UCNPs, which is related to the *ca.* 2.5-fold larger absorption cross section of the former. It is noteworthy that the temperature increase has not reached steady-state for any power density employed. However, the analysis performed regarding the linear relationship between Δ*T*_m_ and *P*_D_ in eqn (1) is still valid because by determining Δ*T* at different times (*e.g.*, 200, 600, 800, and 1200 s), this linear relationship is maintained with the same slopes (Fig. S10, ESI†).

The temporal dependence of the temperature increase, also known as a transient regime before reaching steady-state, can provide additional thermal properties of the nanofluid. The temperature increase with time can described by:^64^

2 Δ*T*(*t*) = Δ*T*_m_(1 − *e*^−*t*/*τ*^), *τ* = *mc*/(*hA*)

where *m* and *c* are the mass and the specific heat capacity of the nanofluid, respectively, *h* is the heat transfer coefficient, and *A* is the thermal contact area through which occurs the heat transfer. This eqn (2) can be obtained from the energy conservation per unit of time, known as the power balance equation, between the excitation cylinder and the surrounding fluid. The assumptions involving this equation are the constant absorption cross-section of the fluid within the excitation cylinder and the energy dissipation are due to the heat transfer and internal energy increase due to the increase of the temperature. Alternatively, by analogy to an electrical circuit, the transient time *τ* can be expressed as *τ* = *RC*, where *R* = 1/(*hA*) is the thermal resistance and *C* = *mc* is the thermal capacitance of the nanofluid.^65^ Thus, fitting eqn (2) to the Δ*T*(*t*) profiles of the nanofluids for all the curves, the values of Δ*T*_m_ and *τ* were obtained (Table S3 and Fig. S11, ESI†). Considering the thermal capacitances, *C*, of the solvent and nanoparticle given in Table S3, ESI,† the thermal resistance, *R*, was calculated for each nanofluid as *R* = *τ*/*C*, yielding values of (7.3 ± 0.1) × 10^5^ K W^−1^ and (6.7 ± 0.1) × 10^5^ K W^−1^ for the oleate capped UCNPs dispersed in cyclohexane and cysteine modified UCNPs dispersed in water, respectively. As in the case of the thermal conductivity of the nanofluid, *κ*_f_, these values calculated for the thermal resistance are tentatively assigned to those of the pure solvent, because the contribution of the nanoparticles is very small compared to that of the solvent. Indeed, these values are consistent with those of the solvents and two orders of magnitude smaller than nanoparticles in air^66–71^ (*e.g.*, KLu(WO_4_)_2_:Ho^3+^, Tm^3+^; 9.5 × 10^7^ K W^−1^).^53^ Because the thermal properties of the nanofluids (thermal conductivity and thermal resistance) are dominated by the solvent, the effects of the organic coating (cysteine modification) on the heat transfer to the environment are inclusive. In situations where the effects of the NPs would be relevant, the difference in contact area of the cysteine modified UCNPs compared to the one of oleate capped UCNPs should affect the thermal resistance as the contact resistance can be considered a dominant factor.^69^

It is noteworthy that the transient behavior of these nanofluids is quantitatively very distinct from the nanofluids composed of coated (lipid bilayers) and uncoated UCNPs (LiYF_4_:Er^3+^/Yb^3+^) suspended in water (H_2_O and D_2_O).^64^ The main difference between these nanofluids is the dynamics of their thermal properties. For instance, the typical transient times (initial temperature increase until constant temperature) for the cysteine-modified UCNPs dispersed in water were *ca.* 350-fold larger than for the UCNPs suspended in water. These different behaviors can be attributed to concentration effects and dispersion *versus* suspension of similar amounts of UCNPs. In the present case, the cysteine-modified UCNPs are soluble in water, hence forming a dispersion with very low concentration. As described, the thermal properties of the nanofluid are dominated by the solvent and the UCNPs have negligible effects. On the other hand, the unmodified UCNPs (either capped or uncapped) were insoluble in water and the nanofluids were investigated as suspensions. Notice that despite the amounts of UCNPs employed in all these experiments being practically the same, the concentrations of the NPs in the suspensions are much higher than those in dispersions, so the thermal behaviors of these nanofluid should be quite distinct. In addition, the motions (diffusion and thermal-diffusion) of the UCNPs in the suspensions are much more restricted than those in diluted dispersions.

Then, it could be inferred that by increasing the concentration of UCNPs dispersed in water, the thermal properties of the nanofluid would become dependent on these UCNPs and novel experiments could be designed to quantify new properties. In this case, as a UCNP leaves the excitation beam, a large amount of solvent replaces it and increases the absorption cross-section, thus increasing the power absorbed from the laser beam, which increases the optical heating and the time to the temperature reaching a steady-state regime. A reverse effect can be devised by a UCNP entering the excitation cylinder. In fact, if the temporal dependence of the temperature increase for the dispersions is affected by the migration of the UCNPs in and out of the excitation cylinder, this could lead to a novel approach to measure the Soret coefficient, *S*_T_, of NPs, which is relevant to opto-thermophoresis, particle separation, and related effects.^72,73^ For instance, at steady-state conditions, the concentration profile of the NPs is described as^74–76^3
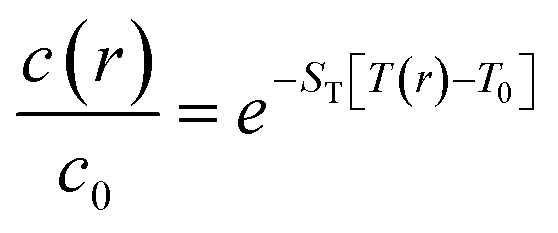
where *c*(*r*) and *T*(*r*) are the concentration of the NPs and the temperature at a radial distance *r* from the excitation cylinder, *c*_0_ and *T*_0_ are the concentration of the NPs and the temperature at bulk. The normalized concentration *c*(*r*)/*c*_0_ can be obtained by probing the UCNPs at a distance *r* with a laser and determining the ratio of the integrated upconversion emission to that at the bulk. Simultaneously, the UC emission spectra at *r* and at the bulk can provide the temperatures *T*(*r*) and *T*_0_. By performing these measurements at several distances *r*, the Soret coefficient *S*_T_ can be estimated from fittings to eqn (3). Alternatively, by setting the conditions where the contribution from the concentration gradient is negligible (low concentrations), the thermal diffusion coefficient *D*_*T*_ can be estimated as^74–76^4
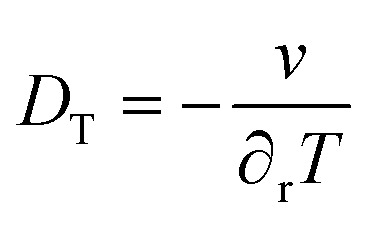
where *v* is the drift (or thermophoretic) velocity of the NPs and ∂_r_*T* is a spatially uniform temperature gradient.^74–76^ The quantity *v* can be determined from the emission spectrum of the UCNPs as shown previously,^77^ whereas the thermometric UCNPs can provide ∂_r_*T*. The consistency of these measurements can then be ascertained by comparing the diffusion coefficient, *D*, derived from *D* = *S*_T_*D*_T_ with that obtained from other techniques.

Thus, these optical techniques provide easy access to the thermal properties of nanoparticles and nanofluids (*e.g.*, thermal resistance and conductivity), with the advantage of being independent of the sample electrical conductivity. Potentially, by probing the dispersed UCNPs the thermophoretic properties of the NPs can also be promptly accessed.

## 4. Conclusions

The ability of LiYF_4_:Yb^3+^/Er^3+^ nanoparticles to be a luminescent thermometer was assessed in two different media. Since oleate capped UCNPs are hydrophobic, cyclohexane was chosen as the solvent. To make the UCNPs water dispersible, a cysteine modification was used by epoxidation of the carbon double bond and opening of the epoxide ring using cysteine. In both nanofluids it was possible to observe an intense Er^3+^ emission when excited at 980 nm. In water, there was a slight increased red emission attributed to a decay from green emitting levels to the red emitting level. Both nanofluids worked as primary thermometers in the temperature range of 290–320 K with a good thermal sensitivity of 1.24% K^−1^, and a thermal uncertainty of 0.8 and 0.5 K for the oleate capped particles dispersed in cyclohexane and cysteine modified particles dispersed in water, respectively. Transient heating curves were obtained, where the water dispersion heated more than the cyclohexane one. Through the steady-state regime of the heating curves, the thermal conductivity of the nanofluids was estimated. The estimated thermal conductivities were 0.123 ± 0.006 W m^−1^ K^−1^ for the oleate capped UCNPs dispersed in cyclohexane, and 0.50 ± 0.07 W m^−1^ K^−1^ for the cysteine modified UCNPs dispersed in water, remarkably close the values of pure solvents, since the nanoparticles had a minor role in the total heating of the system, because of their low concentrations. Using the transient regime of the heating curves, the thermal resistances were calculated, with their values being attributed to the pure solvents because of the diluted dispersions used and could be considered as the first reported values for cyclohexane and water. These tools to determine the nanofluids thermal properties, enlarge the application of luminescence thermometry, allowing the exploitation of the heat transfer processes occurring at the micro and nanoscale.

## Conflicts of interest

There are no conflits to declare.

## Supplementary Material

NA-006-D3NA01114C-s001
