# Supplementary material for: Thermal properties of nanofluids using hydrophilic and hydrophobic LiYF4:Yb/Er upconverting nanoparticles
Source: Nanoscale Adv. 2024 Feb 14;6(5):1486–96. doi: 10.1039/d3na01114c (PMC10898443; doi:10.1039/d3na01114c)
Supplement: NA-006-D3NA01114C-s001 [file NA-006-D3NA01114C-s001.pdf]

Electronic Supplementary Material (ESI) for Nanoscale Advances.
This journal is © The Royal Society of Chemistry 2024

Supplementary information

Thermal properties of nanofluids using hydrophilic and hydrophobic $LiYF_4$:Yb/Er upconverting nanoparticles

João M. Gonçalves,[a,b] Ana R. N. Bastos,[a] Sidney J. L. Ribeiro,[c] L. D. Carlos,[a] Ricardo L. Longo,[d*] José Maurício A. Caiut, [b*] Rute A.S. Ferreira[a*]

[1.a]Department of Physics, CICECO – Aveiro Institute of Materials, University of Aveiro, 3810-193, Aveiro, Portugal
[b]Department of Chemistry, Faculdade de Filosofia, Ciências e Letras, University of São Paulo, 14040-900, Ribeirão Preto, Brazil
[c]Institute of Chemistry, Universidade Estadual Paulista «Júlio de Mesquisa Filho», 14800-060, Araraquara, Brazil
[d]Departamento de Química Fundamental, Universidade Federal de Pernambuco, 50740-540, Recife, PE, Brazil.

***Corresponding authors: caiut@ffclrp.usp.br; rferreira@ua.pt; ricardo.longo@ufpe.br**

I. Structural characterization 2
II. Determination of UCNP and nanofluid properties 4
III. Optical characterization and thermometric characterization of the luminescent thermometers 7
IV. Supplementary tables 13
V. References 16

I. Structural characterization

Powder X-Ray Diffraction experiments were conducted on a Bruker-AXS D2 Phaser diffractometer with Cu Kα radiation (λ = 1.5418 Å) from 10° to 60° at a scanning rate of 5° min^{-1}. Figure S1 shows the obtained XRD patterns for the oleate capped UCNPs which presents narrow peaks in great agreement with the $LiYF_4$ tetragonal phase (JCPDS #81-2254)[1], with no extra peaks observed.

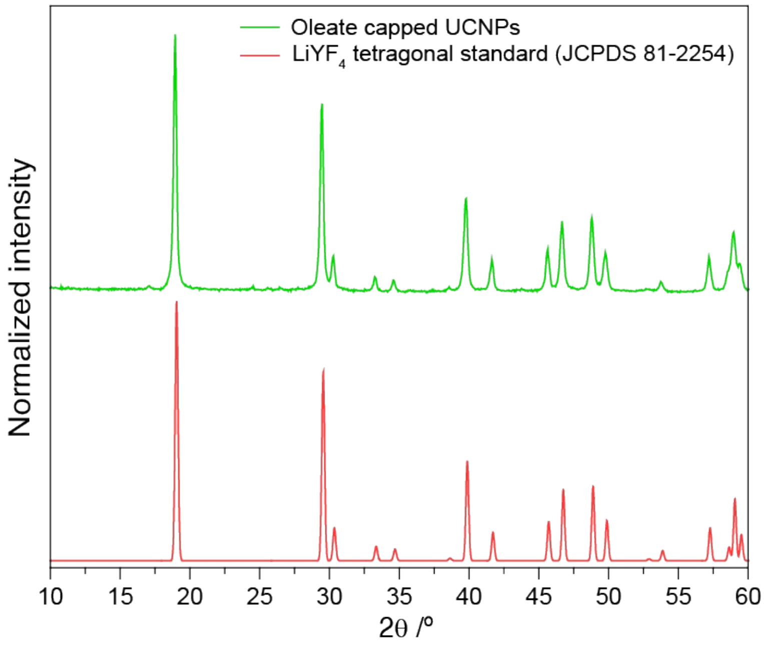

Figure S1 – Powder XRD patterns for the oleate capped UCNPs and $LiYF_4$ tetragonal standard JCPDS 81-2254.

The Fourier Transform Infrared spectroscopy (FTIR) was performed on a Shimadzu IRPrestige-2. All spectra were recorded with 20 scans and 2.0 cm^{-1} resolution in the 4000-400 cm^{-1} window. All samples were prepared as KBr pellets. Figure S2 presents the FTIR spectrum for the oleate capped UCNPs, showing typical oleic acid bands, attributed to stretching of CH_2 groups (at 2920 cm^{-1} and 2850 cm^{-1}) and -COO- groups (at 1560 cm^{-1} and 1460 cm^{-1}). A wide and rather intense band near 3500 cm^{-1} was attributed to OH stretching of unbound oleic acid COOH groups that were not washed away[2–4]. After cysteine modification, C-N and N-H stretching appears at 1146 cm^{-1} and 1208 cm^{-1} and a C=O vibration at 1680 cm^{-1}, characteristic of cysteine[5].

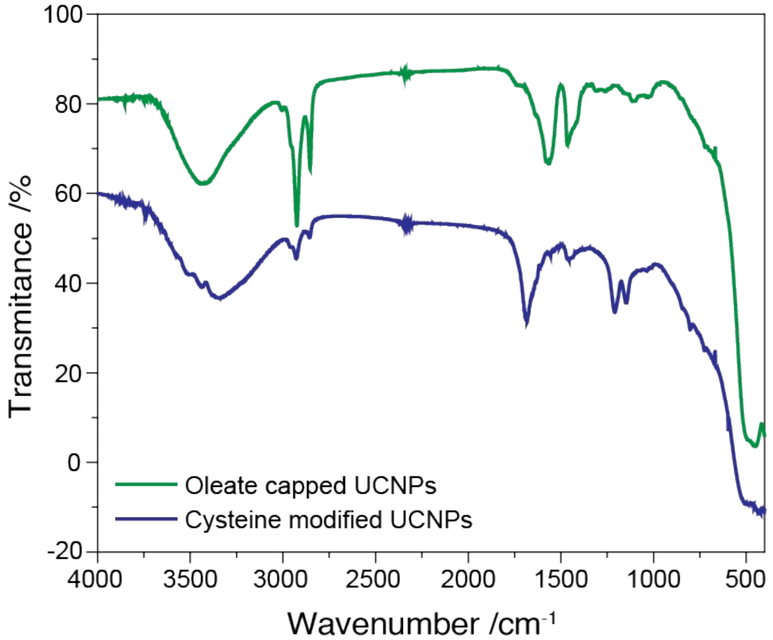

Figure S2 – FTIR spectrum of the oleate capped and cysteine modified $LiYF_4$:Yb3%,Er0.025% nanoparticles.

II. Determination of UCNP and nanofluid properties

The absorption coefficient, α_J (in m^{-1}), of an absorbing species J in the (nano)fluid is

$$\alpha_J = (\ln 10)\frac{A_J}{L} \tag{S1}$$

where A_J is the absorbance of species J using the solvent as the reference and L is the optical pathlength.

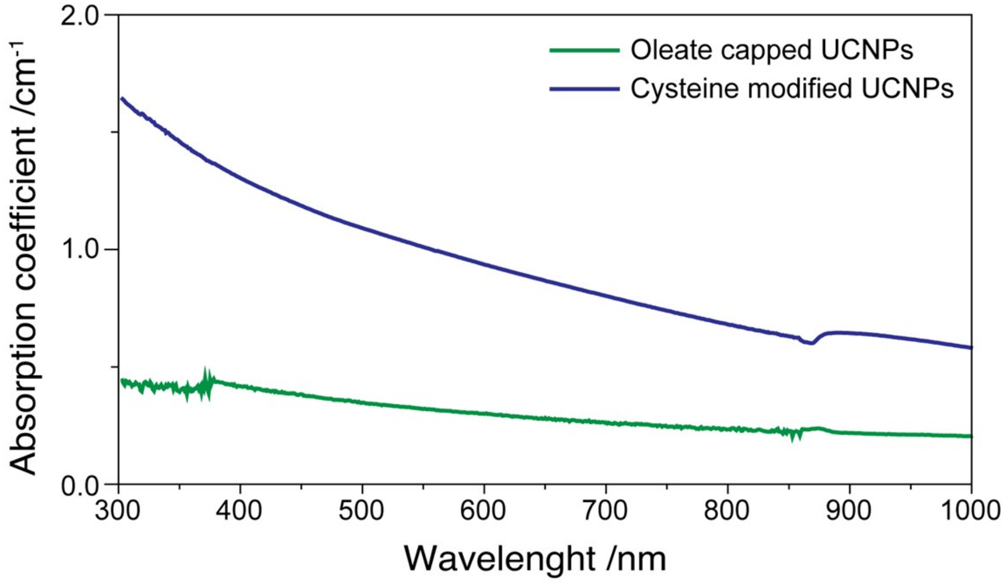

Figure S3 –Visible NIR room temperature absorption spectra of the oleate capped and cysteine modified $LiYF_4$:Yb3%,Er0.025% nanoparticles.

The molar extinction coefficient, $\varepsilon(\lambda)$ (in M^{-1} m^{-1}) at 980 nm is calculated through the following equation:

$$\varepsilon_{UCNP}(980) = \frac{A_J(980)}{L \times c_J} \tag{S2}$$

where $A_J(980)$ is the absorbance at 980 nm, and c_J corresponds to the molar concentration of J-absorbing species.

The absorption cross section, σ_J (in m^2), of a single absorber J in solution is

$$\sigma_J = \frac{\alpha_J}{N_J} \tag{S3}$$

N_J is the number density of J-th absorbers in (# of absorbers-J) m^{-3}. For a solution of absorbers with concentration C_J (in $mg/mL = mg/cm^3$), N_J is

$$N_J(\#absorbers\ m^{-3}) = \frac{C_J}{m_J} \times 10^6 \tag{S4}$$

where m_J (in mg) is the mass of the absorber-J.

Both the oleate capped- and cysteine modified-$LiYF_4$ nanoparticles have a square (or tetragonal) bipyramid shape with small diagonal, d_s, long diagonal d_l, and its volume, V_P, is given by:

$$V_P = \frac{1}{6} d_s^2 d_l \tag{S5}$$

The number of nanoparticles exposed to the laser was calculated from the concentration of nanoparticles in the dispersion, volume of nanoparticles and volume of the cylinder formed by the incident laser on the cuvette. The number of UCNPs, $N_{P,b}$, and of solvent molecules, $N_{S,b}$, exposed to the laser beam are

$$N_{P,b} = N_p A_b L,\ N_{S,b} = N_S A_b L \tag{S6}$$

where N_P (in $\#NPs\ m^{-3}$) is the number density of NPs, N_S (in $\#S\ m^{-3}$) is the number density of solvent, A_b (in m^2) is the area of the laser spot, and L (in m) is the optical path length.

It was also considered that the modification of oleic acid with cysteine did not alter significantly the oleic acid surface area, considered 0.4 nm^2 [6] or the coverage of ligands in the surface of the nanoparticle. From the oleic acid covered area and oleic acid surface area, the number of oleic acid molecules at the surface of the nanoparticles was calculated. With this, it was possible to calculate the mass of oleic acid in each nanoparticle. For the cysteine-modified oleic acid, an analogous procedure was used, but with the molecular weight of the modified oleic acid.

The weight of the cysteine-modified oleic acid was calculated from the sum of oleic acid and cysteine molecular weights. The total mass of the particle plus ligands was calculated from the sum of the mass of one particle and the total mass of ligands in the particles.

The area of the nanoparticle covered with oleic acid must be accounted in the molecular weight of each UCNP. To measure the number of oleic acid molecules attached to each nanoparticle, the thermogravimetric analysis (TGA) was used. Figure S4 shows the TGA profile of the sample, which shows a mass loss of 2.46 % between 280 ^{o}C and 400 ^{o}C [6,7], which represents the loss attributed to the oleic acid bonded to the nanoparticle. Considering the initial mass of 13.4350 mg, the calculated number of molecules of oleic acid was 7.0×10^{17} molecules and using the oleic acid area, the total coverage of the particle was considered 40%, which is in agreement with the literature[6].

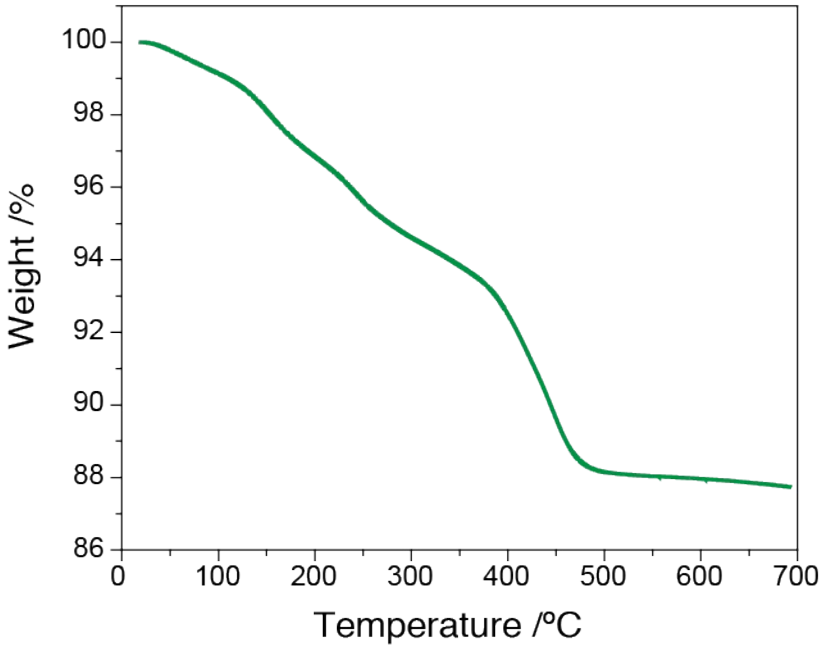

Figure S4 – TGA curve of the oleate capped $LiYF_4$:Yb3%,Er0.025%.

III. Optical characterization and thermometric characterization of the luminescent thermometers

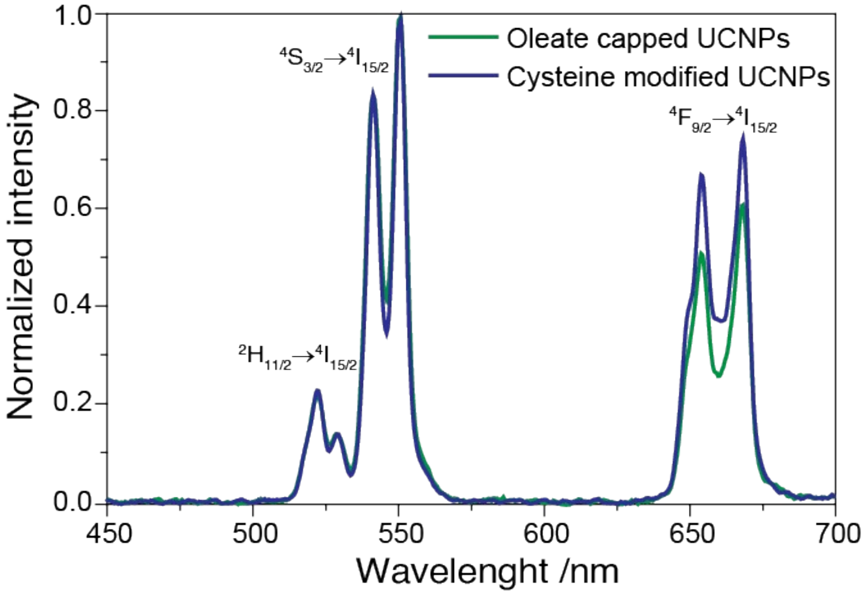

Figure S5 – Normalized emission spectra acquired upon 980 nm excitation (229 W cm^{-2}) for each of the nanofluids. The Er^{3+} transitions are labeled.

To verify the capability of the system to be a primary thermometer, upconversion emission spectra were obtained for temperatures ranging from 293 K to 317 K. The intensity parameter Δ was defined as the intensity ratio between $^2H_{11/2} \rightarrow {}^4I_{15/2}$ and $^4S_{13/2} \rightarrow {}^4I_{15/2}$ transitions of Er^{3+}.

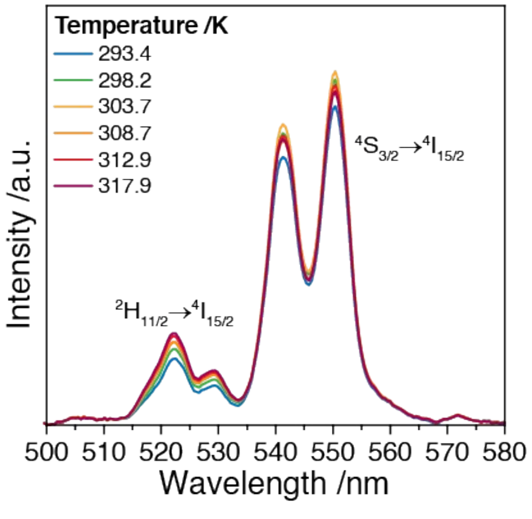

Figure S6 - Temperature-dependence of the upconversion emission spectra of oleate capped UCNPs dispersed in cyclohexane upon irradiation with 980 nm laser (172 W cm^{-2}).

To calculate ΔE, one representative spectrum was plotted, and each one was fitted to a Gaussian distribution. The signal was converted from wavelength to energy units to fit Gaussian functions to the spectrum applying

$$E = \frac{1}{\lambda \times 10^{-7}} \tag{S7}$$

where E is the energy in units of cm^{-1}, and λ is the wavelength in nm units. The Jacobian transformation given below was used to rescale the intensity values as a function of energy units:

$$I(E) = I(\lambda)\frac{d\lambda}{dE} = \frac{I(\lambda)}{E^2 \times 10^{-7}} \tag{S8}$$

where $I(E)$ and $I(\lambda)$ are the intensity as a function of energy and wavelength, respectively. Then, the barycenter of each transition was calculated by a weighted arithmetic mean and the energy difference was calculated. The values calculated were 777 ± 44 cm^{-1} and 775 ± 35 cm^{-1} for the oleate capped and cysteine modified UCNPs, respectively.

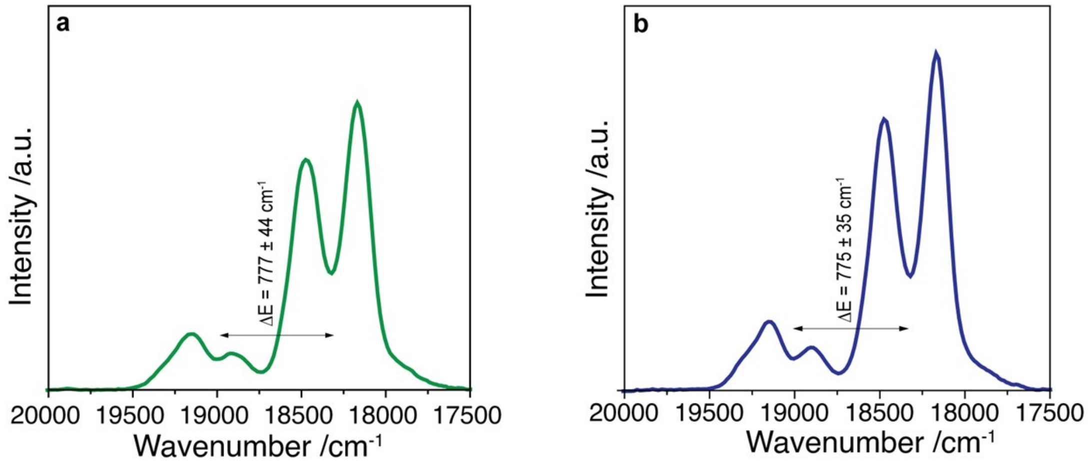

Figure S7 – Emission spectra used to calculate ΔE for (a) oleate capped UCNPs and (b) cysteine modified UCNPs.

To obtain the value of Δ_0, the emission spectra were recorded in different power densities and a linear dependence of the intensity parameter Δ with the laser power density. The intercept of the linear fit was taken as Δ_0, as presented in each one of the graphs and showed in Table S1.

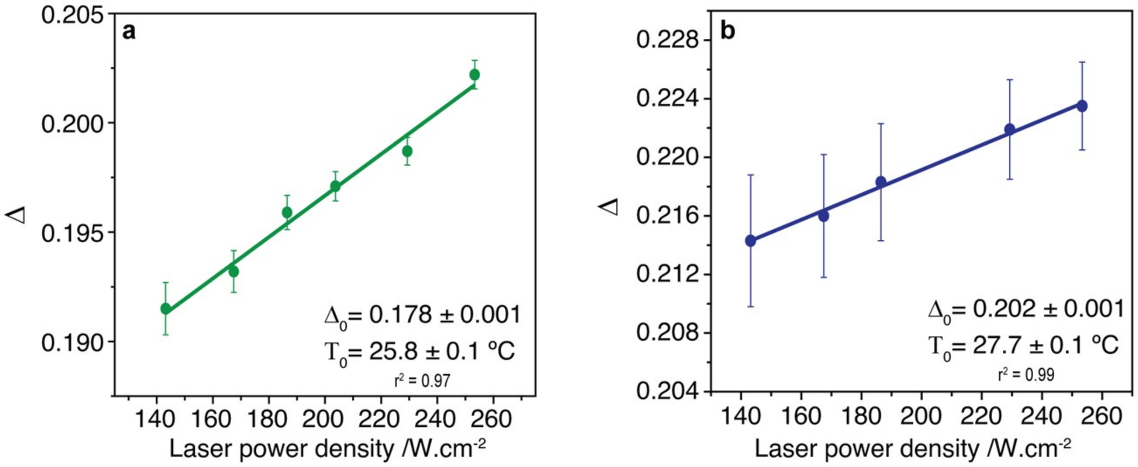

Figure S8 – Dependency of the Δ intensity parameter with the laser power density used to calculate Δ_0 for (a) oleate capped UCNPs and (b) cysteine modified UCNPs.

With all these parameters (Table S1), it was possible to calculate the temperature using the Δ, the following equation was used[8]:

$$\frac{1}{T} = \frac{1}{T_0} - \frac{k_B}{\Delta E}\ln\left(\frac{\Delta}{\Delta_0}\right) \quad \text{(S9)}$$

where k_B is the Boltzmann constant, ΔE is the energy difference between the barycenter of the two emissions, T_0 is the temperature of null laser-induced heating, and Δ_0 is the intensity parameter at T_0.

Relative Thermal Sensitivity and Temperature Uncertainty

To assess the thermometer performance, two figures of merit were calculated, the relative thermal sensitivity and the temperature uncertainty. The relative thermal sensitivity S_r indicates the relative change of Δ per degree of temperature change, and is defined by:

$$S_r = \frac{1}{\Delta}\left|\frac{\partial \Delta}{\partial T}\right| = \frac{\Delta E}{k_B T^2} \quad \text{(S10)}$$

where ΔE is the separation between the thermally coupled energy levels, k_B is the Boltzmann constant, T is the absolute temperature, and Δ is the thermometric parameter. The error related to the sensitivity (δS_r) was derived from the errors of the parameters used in the calculation, as defined by:

$$\delta S_r = \frac{\delta \Delta E}{k_B T^2} \tag{S11}$$

where $\delta\Delta E$ is the error in ΔE. The temperature uncertainty δT is the temperature resolution, *i.e.*, the smallest temperature change that can be detected in a given measurement. The uncertainty of the thermometer temperature is given by:

$$\delta T = \frac{1}{S_r}\frac{\delta\Delta}{\Delta} \tag{S12}$$

where $\delta\Delta/\Delta$ is the relative error in the thermometric parameter.

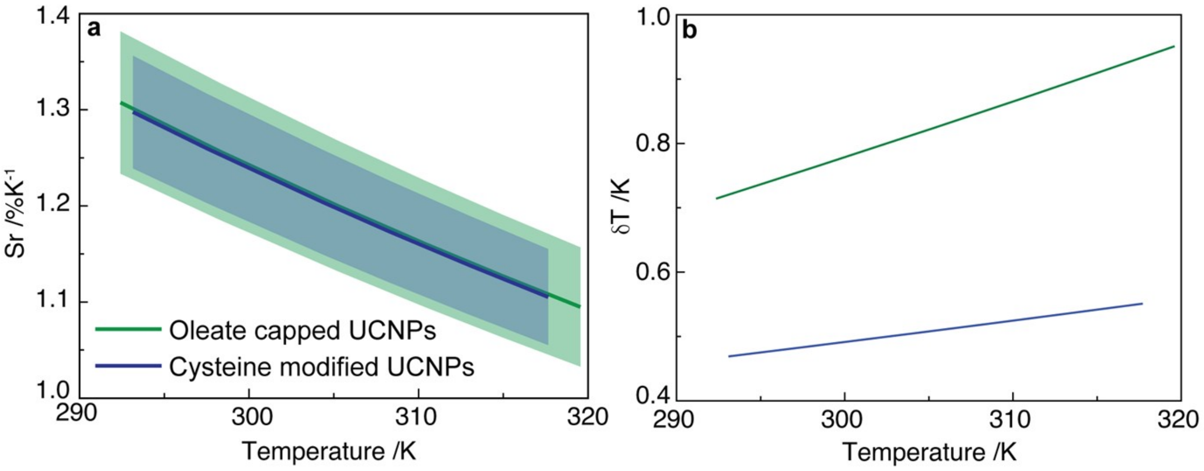

Figure S9 – Thermometric performance of the nanofluids upon 980 nm excitations. (a) Relative thermal sensitivity of the oleate capped UCNPs and cysteine modified UCNPs. The shadowed area corresponds to the respective error calculated using Equation S11. (b) Temperature uncertainty of the nanothermometers.

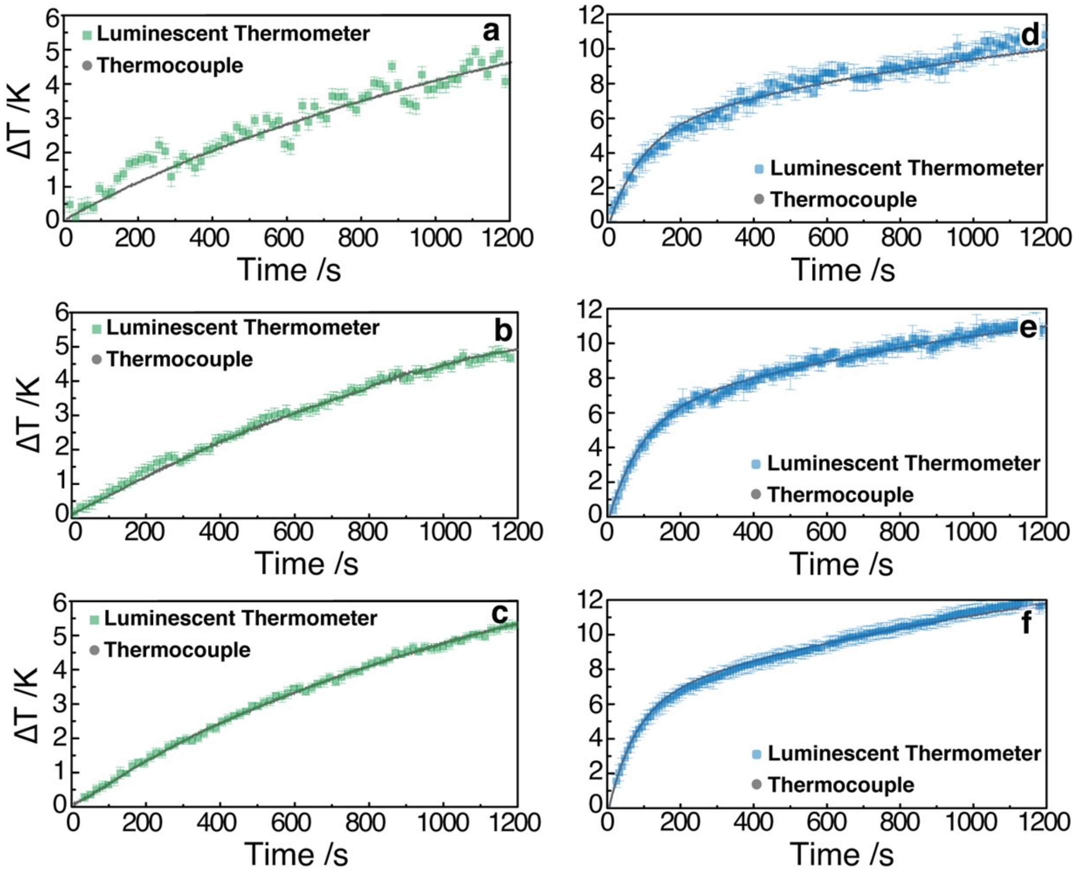

Figure S10 –Temperature profiles of the (a-c) oleate capped UCNPs dispersed in cyclohexane and (d-f) cysteine modified UCNPs dispersed in water, measured by the immersed thermocouple (grey dots) and luminescent thermometer (colored dots). The curves were obtained when the nanofluids were irradiated with a 980 nm laser (200-250 W cm^{-2}). For lower power densities, the luminescent thermometer presented a higher error and scattered.

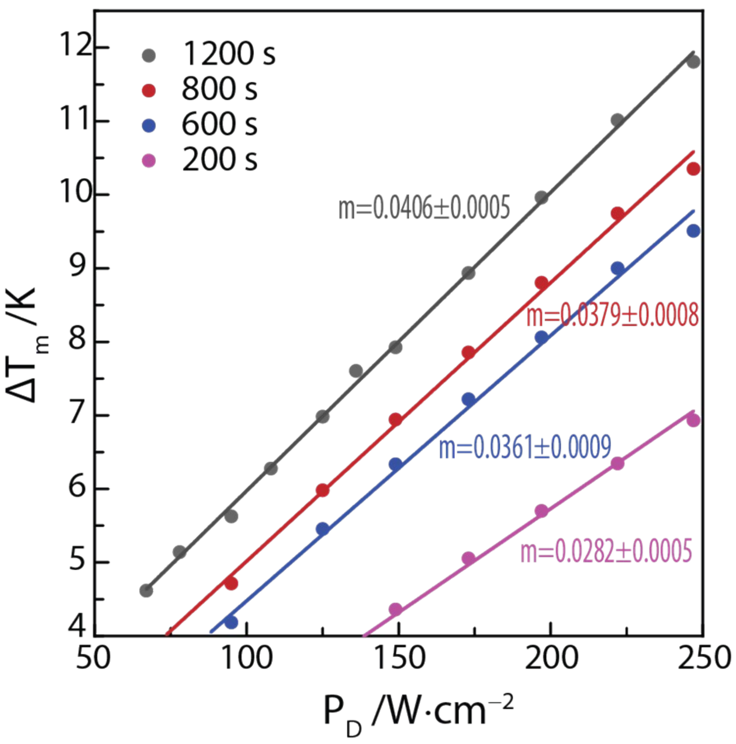

Figure S11 – Relationship between ΔT_m and P_D at different times (e.g., 600, 800, and 1200 s) for the oleate capped UCNPs dispersed in cyclohexane. The solid line represents the dta best linear fit ($r^2 > 0.99$) with slopes analougous slopes (m).

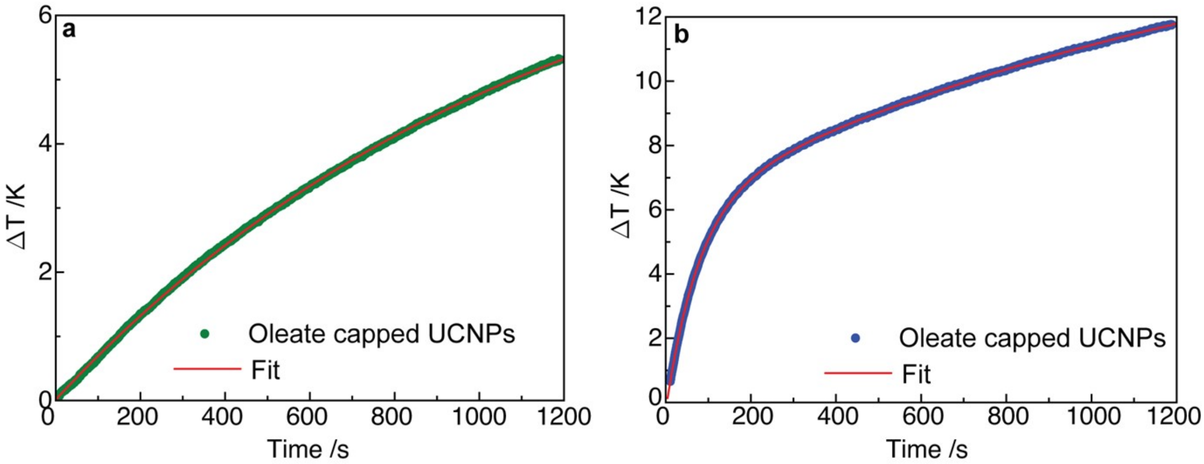

Figure S12 – Temperature profiles of the oleate capped UCNPs dispersed in (a) cyclohexane and (b) cysteine modified UCNPs dispersed in water measured by the thermocouple. The red lines are the fit to the experimental data using Equation (2). The curves were obtained when the nanofluids were irradiated with a 980 nm laser (250 W cm^{-2}).

IV. Supplementary tables

Table S1 Intercept (Δ_0), respective temperature, and slope ($\partial\Delta/\partial P_D$) resulting from the fit of straight lines to the experimental data presented in Figure S8.

Sample	Δ_0	T_0 (±0.1 K)	$\partial\Delta/\partial P_D$ ($\times10^{-5}$ cm^2 W^{-1})
Oleate capped UCNPs	0.178±0.001	298.9±0.1	9.5±0.7
Cysteine modified UCNPs	0.202±0.001	300.8±01	8.5±0.4

Table S2 – Physical properties of oleate capped and cysteine modified $LiYF_4$:Yb3%,Er0.025% nanoparticles.

	Quantity	Value	Unit	Ref
Oleate capped UCNPs	Concentration	4	g L^{-1}	
	Molar Mass ($LiYF_4$:3% Yb/0.025% Er)	174.34	g mol^{-1}	
	Size long diagonal	94 ± 15	nm	
	Size short diagonal	65 ± 6	nm	
	Volume one UCNP	6.62 ± 2.28	10^{-17} cm^3	
	Density	3.995	g cm^{-3}	9
	Mass one UCNP	1.5 ± 0.3	10^{-13} mg	
	Particle surface area	8000	nm^2	
	Area covered by oleic acid	3200	nm^2	
	Oleic acid surface area	0.4	nm^2	
	Oleic acid molecule weight	4.69	10^{-25} mg	
	Oleic acid mass in one particle	3.75	10^{-21} mg	
	Mass of one oleate capped UCNP	1.61	10^{-13} mg	
	Beam area	8.01 ± 0.01	10^{-9} m^2	
	Number of UCNP exposed to laser	2.0 ± 0.4	10^9	
	Absorption cross section	9.3 ± 0.4	10^{-19} m^2	
	Geometrical correction factor	1.39 ± 0.25		
	Equivalent radius	2.11±0.21	10^{-8} m	
Cysteine Modified UCNPs	Concentration	4	g L^{-1}	
	Molar Mass($LiYF_4$:3% Yb/0.025% Er)	174.34	g mol^{-1}	
	Size long diagonal	85 ± 12	nm	
	Size short diagonal	46 ± 5	nm	
	Volume one UCNP	3.00 ± 1.07	10^{-17} cm^3	
	Density	3.995	g cm^{-3}	9
	Mass one UCNP modified with cysteine	1.62	10^{-13} mg	
	Particle surface area	8000	nm^2	
	Area covered by oleic acid modified with cysteine	3200	nm^2	
	Oleic acid modified with cysteine surface area	0.4	nm^2	
	Oleic acid modified with cysteine molecule weight	6.70	10^{-25} mg	
	Oleic acid modified with cysteine mass in one particle	5.36	10^{-21} mg	
	Beam area	8.01	10^{-9} m^2	
	Number of UCNP exposed to laser	2.0 ± 0.4	10^9	
	Absorption cross section	2.3 ± 0.1	10^{-18} m^2	
	Geometrical correction factor	1.66 ± 0.19		
	Equivalent radius	2.11	10^{-8} m	

Table S3. Properties of the oleate capped and cysteine modified $LiYF_4$:Yb3%,Er0.025% nanoparticles for the thermal resistance calculations.

	**Oleate capped UCNPs dispersed in cyclohexane**	**Cysteine Modified UCNPs dispersed in water**
ΔT_m **[K]**	8.23±0.01	16.56±0.04
τ **[s]**	80±2	226±3
**UCNPs mass [×10^{-10} kg]**	3.2±0.1	3.2±0.1
**Solvent mass [×10^{-8} kg]**	6.240±0.008	7.997±0.004
**Cysteine mass [×10^{-17} kg]**	-	1.06±0.04
c_{LiYF_4} **[J K^{-1} kg^{-1}]**	790[10]	
$c_{cysteine}$ **[J K^{-1} kg^{-1}]**	138.2[11]	
$c_{cyclohexane}$ **[J K^{-1} kg^{-1}]**	1749.3[12]	
c_{water} **[J K^{-1} kg^{-1}]**	4182[13]	
C **[×10^{-4} J K^{-1}]**	1.094±0.001	3.350±0.003
R **[×10^{5} K W^{-1}]**	7.31±0.1	6.75±0.1

V. References

(1) Mahalingam, V.; Vetrone, F.; Naccache, R.; Speghini, A.; Capobianco, J. A. Colloidal Tm3+/Yb3+-Doped LiYF4Nanocrystals: Multiple Luminescence Spanning the UV to NIR Regions via Low-Energy Excitation. *Adv. Mater.* **2009**, *21* (40), 4025–4028. https://doi.org/10.1002/adma.200901174.

(2) Ramírez-García, G.; Honorato-Colin, M. Á.; De la Rosa, E.; López-Luke, T.; Panikar, S. S.; Ibarra-Sánchez, J. de J.; Piazza, V. Theranostic Nanocomplex of Gold-Decorated Upconversion Nanoparticles for Optical Imaging and Temperature-Controlled Photothermal Therapy. *J. Photochem. Photobiol. A Chem.* **2019**, *384*. https://doi.org/10.1016/j.jphotochem.2019.112053.

(3) Chen, Z.; Chen, H.; Hu, H.; Yu, M.; Li, F.; Zhang, Q.; Zhou, Z.; Yi, T.; Huang, C. Versatile Synthesis Strategy for Carboxylic Acid-Functionalized Upconverting Nanophosphors as Biological Labels. *J Am Chem Soc* **2008**, *130* (10), 3023–3029. https://doi.org/10.1021/ja076151k.

(4) Yu, S.; Gao, X.; Jing, H.; Zhao, J.; Su, H. A Synthesis and Up-Conversional Photoluminescence Study of Hexagonal Phase NaYF4:Yb,Er Nanoparticles. *CrystEngComm* **2013**, *15* (46). https://doi.org/10.1039/c3ce41857j.

(5) Wei, Z.; Sun, L.; Liu, J.; Zhang, J. Z.; Yang, H.; Yang, Y.; Shi, L. Cysteine Modified Rare-Earth up-Converting Nanoparticles for in Vitro and in Vivo Bioimaging. *Biomaterials* **2014**, *35* (1), 387–392. https://doi.org/10.1016/j.biomaterials.2013.09.110.

(6) Rojas-Gutierrez, P. A.; DeWolf, C.; Capobianco, J. A. Formation of a Supported Lipid Bilayer on Faceted LiYF4:Tm3+/Yb3+Upconversion Nanoparticles. *Part. Part. Syst. Charact.* **2016**, *33* (12), 865–870. https://doi.org/10.1002/ppsc.201600218.

(7) Bogdan, N.; Vetrone, F.; Roy, R.; Capobianco, J. A. Carbohydrate-Coated Lanthanide-Doped Upconverting Nanoparticles for Lectin Recognition. *J. Mater. Chem.* **2010**, *20* (35). https://doi.org/10.1039/c0jm01617a.

(8) Balabhadra, S.; Debasu, M. L.; Brites, C. D. S.; Ferreira, R. A. S.; Carlos, L. D. Upconverting Nanoparticles Working As Primary Thermometers In Different Media. *J. Phys. Chem. C* **2017**, *121* (25), 13962–13968. https://doi.org/10.1021/acs.jpcc.7b04827.

(9) Shand, W. A. Single Crystal Growth and Some Properties of LiYF4. *J. Cryst. Growth* **1969**, *5* (2), 143–146. https://doi.org/https://doi.org/10.1016/0022-0248(69)90028-1.

(10) Aggarwal, R. L.; Ripin, D. J.; Ochoa, J. R.; Fan, T. Y. Measurement of Thermo-Optic Properties of Y3Al5O12, Lu3Al5O12, YAIO3, LiYF4, LiLuF4, BaY2F8, KGd(WO4)2, and KY(WO4)2 Laser Crystals in the 80–300K Temperature Range. *J. Appl. Phys.* **2005**, *98* (10), 103514. https://doi.org/10.1063/1.2128696.

(11) Foces-Foces, C.; Roux, M. V.; Notario, R.; Segura, M. Thermal Behavior and Polymorphism in Medium–High Temperature Range of the Sulfur Containing Amino Acids l-Cysteine and l-Cystine. *J. Therm. Anal. Calorim.* **2011**, *105* (3), 747–756. https://doi.org/10.1007/s10973-011-1302-6.

(12) Zhou, Y.; Liu, J.; Penoncello, S. G.; Lemmon, E. W. An Equation of State for the Thermodynamic Properties of Cyclohexane. *J. Phys. Chem. Ref. Data* **2014**, *43* (4), 43105. https://doi.org/10.1063/1.4900538.

(13) Haynes, W. M.; Lide, D. R.; Bruno, T. J. *CRC Handbook of Chemistry and Physics*; CRC Press: Boca Raton, FL, 2017.
